# Pore-Scale Analysis and Visualization of Tertiary Cationic Surfactant Flooding in a Complex Carbonate

**DOI:** 10.1021/acsomega.5c06863

**Published:** 2025-10-23

**Authors:** Hussain M. AlZahrani, Branko Bijeljic, Rukuan Chai, Martin J. Blunt

**Affiliations:** Department of Earth Science and Engineering, 4615 Imperial College London, London, South Kensington SW7 2AZ, United Kingdom

## Abstract

We investigate the pore-scale mechanisms of displacement using cationic surfactant dodecyltrimethylammonium bromide (DTAB) flooding as a tertiary enhanced oil recovery (EOR) technique in a complex carbonate rock using high-resolution X-ray microcomputed tomography (micro-CT). A carbonate sample was initially altered to be oil-wet through contact with crude oil, followed by sequential brine and surfactant injection to evaluate the impact on oil displacement and investigate oil recovery mechanisms. We used a concentration of DTAB above the critical micelle concentration, CMC, with no cosolvent. Image-based pore-scale analysis of fluid occupancy, wettability alteration, contact angles, interfacial areas, capillary pressure, and fluid connectivity was performed. The contact angles, mean curvatures, and capillary pressures indicated a transition from oil-wet to a mixed-wet state when we switch from brine to surfactant injection. The results indicate that DTAB flooding reduced interfacial tension and altered wettability, leading to increased oil recovery, particularly from small pores and throats, where brine flooding was ineffective. The results were compared to those of a previous study of secondary DTAB flooding. We hypothesize that wettability alteration was caused by competitive adsorption and hydrophobic interactions, promoting oil detachment from the rock surface. This study demonstrates that DTAB can enhance oil recovery in carbonate formations without requiring ultralow interfacial tension, making it a cost-effective alternative for complex reservoirs.

## Graphical abstract

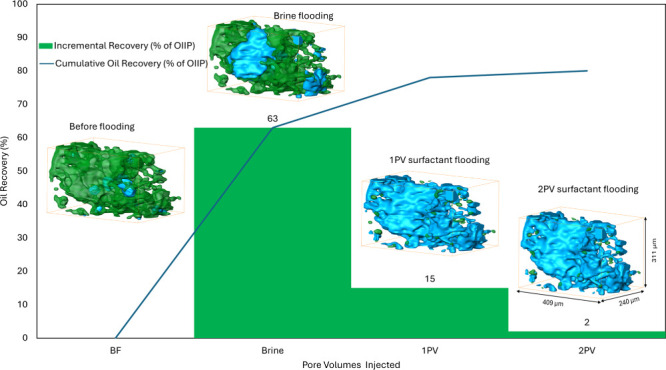

## 1 Introduction

Carbonate reservoirs, which account for 50–60% of the world’s oil and gas reserves, are essential for meeting global energy demand, particularly in the Middle East, where they are the principal oil-bearing formations.^1–3^ The complex nature of these reservoirs due to geological processes and oil wettability characteristics presents challenges in hydrocarbon extraction, with primary and secondary recovery methods typically yielding only a fraction of the original oil in place. In this paper, tertiary cationic surfactant (DTAB) flooding will be studied as an enhanced oil recovery (EOR) technique that offers distinct advantages in complex carbonate systems, including additional recovery caused by a wettability alteration. In mature reservoirs where traditional EOR methods have limitations, tertiary cationic surfactant flooding can substantially enhance oil recovery.^4–7,7^ These surfactants improve oil displacement and recovery by reducing interfacial tension and shifting rock wettability toward a more water-wet state, effectively mobilizing oil trapped within the pore space.^8–11^

The oil-wet nature of carbonate rocks is widely attributed to the selective adsorption of carboxylate molecules from crude oil onto calcite surfaces.^12^ Cationic surfactants have been found to be more effective in enhancing oil recovery than other types of surfactants, primarily because of their ability to alter wettability.^13–17^ This wettability alteration is generally understood to occur through the formation of ion pairs between negatively charged carboxylate species and positively charged cationic surfactants.^11,17–19^ The wettability alteration mechanism has been extensively studied using various techniques, including chromatographic tests, NMR, FTIR, thermogravimetric analysis, and atomic force microscopy.^20–23^ Despite their effectiveness in characterizing wettability changes, these methods cannot provide the detailed pore-scale visualization and analysis necessary to fully understand the underlying interactions between the fluids and the rock.

Microcomputed tomography (micro-CT) is an X-ray imaging technique that allows for nondestructive, three-dimensional visualization of porous media and the fluids within them. By combining high-pressure and high-temperature apparatus with imaging, we can provide detailed microscale analysis of both fluid flow behavior and wettability at the microscale.^24,25^ Its high-resolution imaging capability has made it an essential tool for investigating complex structures within porous media, contributing significantly to our understanding of subsurface processes. From pore geometry and connectivity to derived parameters such as permeability and capillary pressure, micro-CT provides crucial insights into the complex interplay between fluid–fluid and fluid–solid matrices, including the characterization of wettability.^26,27^

Micro-CT has been widely used to investigate the fundamental oil recovery mechanisms in surfactant flooding in various rock types, including carbonates and sandstones.^28–30^ Unlike previous research that focused on achieving ultralow interfacial tension (IFT) (below 10^–^^2^ mN/m) through cosolvent-stabilized anionic microemulsions, our research employs DTAB without the addition of cosolvents, making it more economically viable for use in complex carbonate rocks. The measured IFT in our study is 6.3 mN/m, which is considerably higher than the ultralow values for the other types of surfactant used in previous work.^28–30^ Rather than aiming for an ultralow IFT to completely displace oil with the first pore volume injected, our focus is on understanding the recovery mechanism, particularly the role of wettability and IFT in enhancing oil recovery.

This study employs a comprehensive approach, integrating coreflooding experiments, micro-CT imaging, and image analysis, to investigate tertiary flooding with DTAB in carbonate rock. An oil-wet state was established by saturating the sample with crude oil at an elevated temperature. Brine was then injected, followed by surfactant injection as a tertiary recovery method. Both brine and surfactant were injected at a constant flow rate, simulating a capillary-dominated regime. High-resolution micro-CT images were acquired at various stages to analyze fluid saturation, the spatial distribution of in situ contact angles, pore occupancy, specific interfacial areas, and wettability. Interfacial tension measurements were also conducted for both oil–brine and oil-surfactant systems. The objective of our work is to elucidate the relationship among surfactant-induced wettability changes, contact angles, curvatures (used to determine capillary pressure), fluid distribution, and overall oil recovery, ultimately contributing to a deeper understanding of the mechanism of surfactant-enhanced oil recovery in complex carbonate reservoirs.

Building upon our previous work on secondary surfactant flooding,^31^ the current study advances this research with a focus on the more commonly applied tertiary surfactant flooding. The work provides a detailed exploration of the mechanisms involved in oil mobilization and displacement. Unlike secondary recovery, which recovers more readily accessible oil, tertiary surfactant flooding targets residual oil that remains trapped in the reservoir after secondary recovery methods have been exhausted. This work expands the understanding of DTAB-induced wettability alteration using high-resolution micro-CT imaging to examine how surfactants affect fluid distribution, oil recovery, and wettability at the pore scale. To ensure a direct and meaningful comparison with our earlier findings, we maintained identical experimental conditions and materials compared to our previous study.^31^

The novel contribution of this study lies in exploring the effects of a cationic surfactant (DTAB) without a cosolvent in carbonate rocks, demonstrating its ability to significantly enhance oil recovery without relying on the ultralow interfacial tension values typically achieved by microemulsions stabilized with cosolvents. This is distinct from previous studies and demonstrates an easier, lower-cost option for surfactant EOR. Furthermore, this research provides a detailed visualization and comprehensive analysis of wettability alteration at the pore level, offering new insights into this key recovery mechanism.

## 2 Materials and Methods

### 2.1 Rock Samples and Fluid Properties

In this study, we used Estaillades carbonate, which is composed of 97 wt % calcite with a small amount of dolomite. The rock is known for its complex pore structure: It is a bioclastic grainstone, from a region near Oppede, France,^32^ characterized by a medium to coarse grain size and moderate sorting. It features notable intergranular macro-porosity, along with abundant microporosity present within the bioclasts. We selected a cylindrical sample with a diameter of 6 mm and a length of 14 mm. Using a helium porosimeter (Micromeritics AccuPyc II 1340), the sample’s porosity was measured to be 30.1 ± 0.1%. The micro-CT image porosity, as measured through segmentation, was found to be lower than the total helium porosity. This is because micro-CT imaging captures only the macro-porosity, leaving out the finer microporosity within the rock grains, which cannot be visualized using the resolution of the instrument. The absolute permeability was determined to be 0.11 ± 0.03 Darcy, calculated from Darcy’s law by measuring the pressure drop during brine injection.

Brine was prepared in the laboratory by dissolving various pure salts (supplied by Sigma-Aldrich) in deionized water in specific ratios, resulting in a synthetic brine solution with a total salinity of 44,920 ppm, as shown in Table 1. This salinity was selected based on findings from several studies indicating that, at this concentration, DTAB remains fully soluble in brine without precipitation. Moreover, this salinity level falls within the range typical of some Middle Eastern oil reservoirs, ensuring effective surfactant performance under the experimental conditions.^33–36^

**Table 1:** Brine Chemical Composition

salts	KCl	CaCl_2_	MgCl_2_	NaCl	NaHCO_3_	total
concentration (g/L)	0.477	9.5	3.642	31.178	0.124	44.92

Crude oil was initially used to modify the rock’s wettability to match the original reservoir conditions, which is oil-wet.^37^ The properties of the crude oil are summarized in Table 2. Once the wettability alteration process was achieved, the crude oil was replaced with decane (purity ≥99.0%, Sigma-Aldrich) as the oil phase in the experiment. To provide distinct X-ray attenuation between the oil and brine/surfactant phases in the pore-scale images, decane was doped with 20% 1-iododecane.

**Table 2:** Crude Oil Properties

density at 21 °C (kg/m^3^)	saturates (wt %)	aromatics (wt %)	resins (wt %)	asphaltenes (wt %)	total acid number (mg KOH/g)	total base number (ppm)
830 ± 5	55.25	38.07	6.22	0.46	0.24	356

We employed a cationic surfactant with trade and IUPAC name DTAB and dodecyl trimethylammonium bromide, respectively. The chemical formula for DTAB is C_15_H_34_BrN, and it has a molecular mass of 308.34 g/mol. This surfactant was sourced from Sigma-Aldrich. We prepared the brine solution with a DTAB concentration of 3.24 × 10^–2^ M, which is well above the surfactant’s critical micelle concentration (CMC) of 1.4 × 10^–2^ M (at 25 °C).^11,15^

The interfacial tensions between the experimental fluids—brine, surfactant (a mixture of brine and 10 g/L surfactant), and oil (composed of 80 wt % decane and 20 wt % iododecane)—were measured using the pendant drop technique. This method applies the Young–Laplace equation to images captured under ambient conditions with a Ramé-Hart instrument (model 590).^38,39^ Additionally, the density and viscosity of the brine, surfactant, and oil were measured under the same conditions using Anton Paar viscometers (SVM 300). A summary of the thermophysical properties of the fluids used in this experiment is provided in Table 3.

**Table 3:** Density, Viscosity, and Interfacial Tension of Different Fluids Used in the Experiment

fluid type	density (kg/m^3^)	kinematic viscosity (m^2^/s) × 10^–6^	dynamic viscosity (mPa·s)	interfacial tension (mN/m)
brine (B)	1029	0.967	0.995	oil–brine 43.8
oil (O)	815	1.318	1.099	
surfactant (brine and 10 g/L surfactant) (S)	1031	1.107	1.141	oil–surfactant 6.3

### 2.2 Experimental Apparatus and Wettability Alteration

The experimental apparatus used to alter wettability and the flow experiment is illustrated in Figure 1. The rock sample was first cleaned and dried in a vacuum oven at 60 °C for 2 days to ensure it was thoroughly dehydrated. Following this, the sample was placed inside a cylindrical Viton sleeve with the top and bottom ends of the sleeve secured to ceramic disks and metal end pieces. These metal pieces were then connected to the inlet and outlet flow lines using nuts and ferrules. The entire assembly was housed within a Hassler-type flow cell made from carbon fiber, which is both X-ray transparent and capable of withstanding high pressures and temperatures. The flow cell was positioned in the chamber of a micro-CT scanner and was connected to syringe pumps through PEEK tubes and valves. These pumps were responsible for regulating both the flow rate and the pressure within the system.

**Figure 1:**
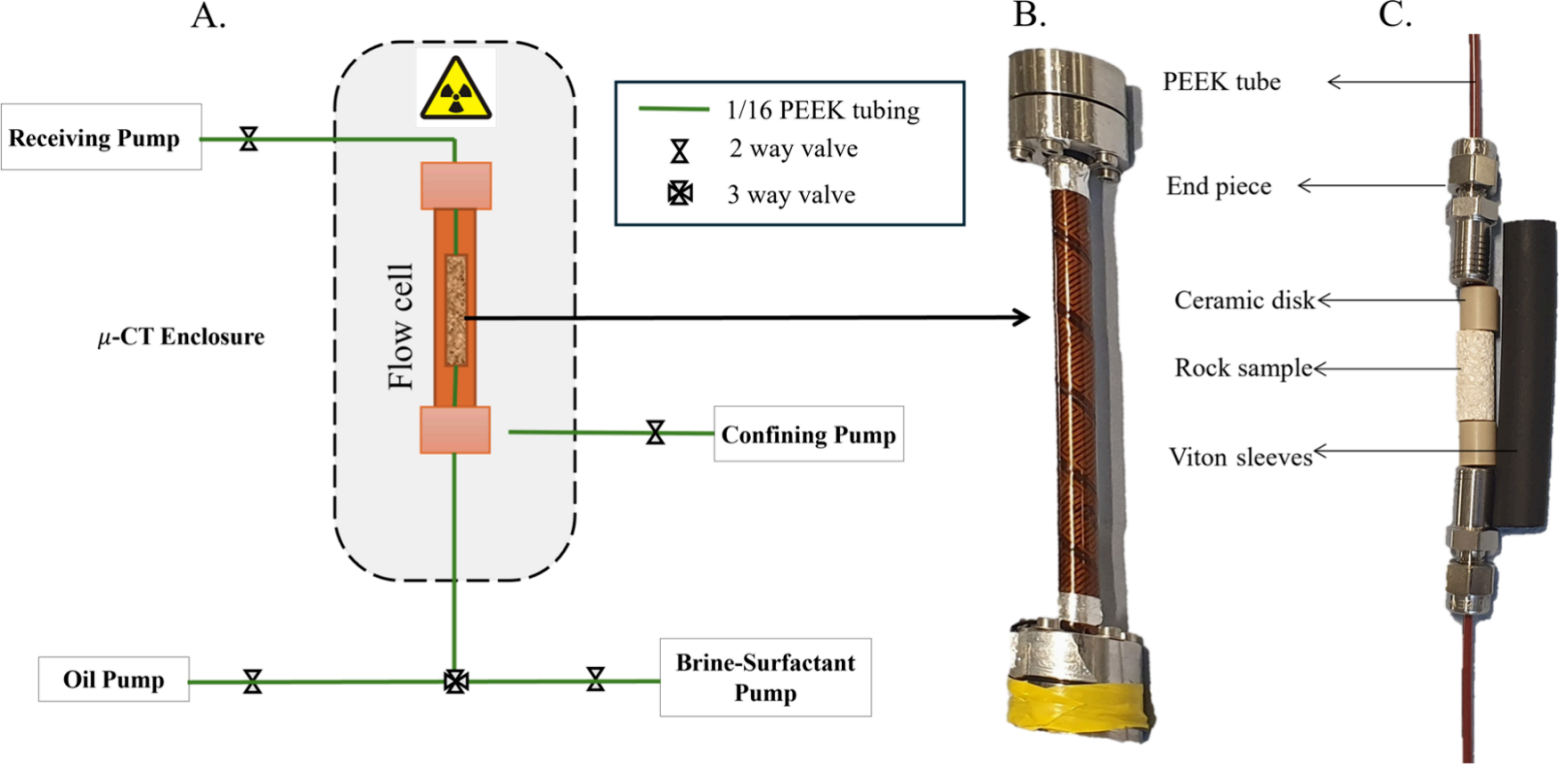
Experimental apparatus used in the experiment. (A) Syringe pumps and flow lines connected to the micro-CT enclosure in the shaded area. (B) Core holder assembly. (C) Rock sample before being fitted in the Viton sleeve with ceramic disks and end pieces all connected through PEEK tubing.

To simulate oil reservoir conditions, the rock sample’s surface wettability was altered prior to the experiment. This was achieved by exposing the rock sample to crude oil under an elevated temperature and pressure, which promotes the adsorption of organic surface-active materials from the crude oil onto the rock, making it oil-wet. The following steps were followed during the process.

1. A confining pressure of 1 MPa was applied between the sample’s Viton sleeve and the inner surface of the core holder.
2. A baseline scan of the dry sample was conducted, referred to as the ″dry scan″. This served as the reference image for the segmentation of rock grains and the identification of the pore space.
3. Brine injection was initiated by introducing 80 pore volumes into the sample from the bottom, beginning with a flow rate of 0.01 mL/min, which was gradually increased to 0.8 mL/min. The injection process was conducted under a pore pressure of 1 MPa and confining pressure of 2 MPa and ambient temperature, aiming to displace air from the sample and establish conditions that mimic the reservoir environment before oil migration. The sample’s absolute permeability was subsequently measured.
4. Drainage commenced with the injection of 100 pore volumes of crude oil, gradually increasing the flow rate from 0.01 to 0.5 mL/min to reach the initial water saturation (*S*_wi_), while maintaining the same pressure and temperature as the brine injection phase.
5. Finally, the flow cell assembly was taken from the micro-CT and placed in an oven at 80 °C with a 2 MPa back pressure and 3 MPa confining pressure for wettability alteration, during which crude oil was injected at a constant rate of 1 μL/min for 4 weeks. After 2 weeks, the flow direction was reversed to ensure that crude oil contacted all the surfaces of the sample.

### 2.3 Brine and Surfactant Flooding

The flooding experiment was carried out in tertiary mode, starting with the injection of one pore volume of brine for secondary oil recovery followed by two pore volumes of surfactant for tertiary recovery. The process took place at room temperature with a confining pressure of 2 MPa and a pore pressure of 1 MPa. The choice of ambient conditions is based on the understanding that pressure has no significant direct influence on surfactant flooding, whereas elevated temperature is known to enhance the performance by lowering interfacial tension (IFT). Accordingly, the system was evaluated under the lowest practical conditions to better evaluate DTAB flooding.^33,34,40^ The procedure for brine and surfactant flooding included the following steps.

1. After completing the wettability alteration as outlined in Section 2.2, the sample was placed back into the micro-CT scanner. Then, 90 pore volumes of decalin were injected to displace the crude oil and prevent asphaltene precipitation during decane injection, as suggested by prior studies.^41^
2. Oil (doped decane) was injected for 100 pore volumes, with the flow rate gradually increased from 0.01 to 0.9 mL/min while ensuring that the differential pressure between the sample’s inlet and outlet remained below 200 kPa.
3. Micro-CT imaging was performed to capture the initial oil-in-place conditions.
4. One pore volume of brine was injected, followed by two pore volumes of surfactant at a constant flow rate of 10 μL/min, achieving a capillary number (Ca = μ*q*/σ, where σ is the oil–water interfacial tension, μ is the viscosity of the aqueous phase, and *q* is the Darcy velocity of the injected surfactant solution) of 1.15 × 10^–^^6^, which resulted in capillary-dominated flow. High-resolution micro-CT images were taken after each pore volume injection to observe the distribution of fluids within the pore space. The confining pressure and pore pressure for the oil, brine, and surfactant flooding were 2 and 1 MPa, respectively.

### 2.4 Image Acquisition, Processing, and Segmentation

An FEI HeliScan micro-CT scanner (Thermo Fisher) was used to acquire high-resolution 3D images after each injection stage where each acquisition took 24 h to acquire, with the fluids inside the rock allowed to equilibrate for 1 h before imaging. The X-ray source was set to a tube voltage of 100 keV and a current of 70 μA, generating a target current of 55 μA, while the sample rotated 360° during scanning to generate a 3D tomogram. The resulting images had a resolution of 2.8 μm per voxel, with a size of 1936 × 1988 × 3755 voxels, achieved with an exposure time of 1.35 s and a total of 3754 projections. This resolution allows for accurate imaging of macro-pores and larger throats (restrictions between wider pores), which are critical for understanding fluid distribution and recovery mechanisms at the pore scale. However, the system cannot reliably resolve micropores smaller than 2.8 μm in diameter. We assume that the unresolved porosity remained brine saturated. As a result, the analysis focuses on macro-porosity and larger pore structures, which are key to understanding fluid flow and oil recovery. These high-resolution scans were essential for characterizing properties such as curvature, fluid–fluid, and fluid–solid interfacial areas, and contact angles. After the images were acquired at different stages, they were reconstructed into 3D grayscale images representing the rock and its fluid content.

Image processing followed, beginning with the removal of irrelevant regions such as the Viton sleeve and core holder. Next, all images from the different flow stages were registered to the dry image (reference image) to maintain a consistent orientation. A nonlocal means filter was applied to enhance the image quality by reducing noise and enabling more accurate segmentation, preserving phase boundaries.^42^

The seeded watershed algorithm was used for phase segmentation to address misclassification issues at the fluid–fluid and fluid–solid interfaces, particularly due to the partial volume effect.^43,44^ It involved seeding homogeneous regions of each phase and then growing the seeded regions until they met phase boundaries.^45–47^ This segmentation method was used to segment the macropore space, as micropores were too small to be captured at this resolution. The segmented images were then analyzed to determine the oil saturation profile. Furthermore, contact angles and curvatures at the interfaces between the oil, the aqueous phase, and the rock were measured.

### 2.5 Rock Wettability Characterization Methods

The geometric contact angle is a useful indicator of the wettability, typically measured under static conditions as a single value on a treated external surface that might not necessarily represent the actual value encountered inside the pore space during a flow experiment. To address this, we employed *in situ* measurements of the contact angle’s spatial distribution between fluids within the pore space using the segmented images. This involved fitting smoothed surfaces to the oil/brine interfaces from segmented 3D images and determining their points of intersection with the rock surface by using perpendicular vectors. An automated algorithm enabled the acquisition of hundreds of thousands of contact angle values.^48–51,47^

In addition to the contact angle, the curvature serves as another important indicator of rock wettability. To measure curvature, the interfaces between the oil and aqueous phases were first extracted using a quadratic equation and then smoothed using a modified Gaussian method.^52^ This smoothing technique was applied to the oil–brine/surfactant interfaces obtained from the segmented images. The principal curvature values and their respective directions were determined by calculating the eigenvalues and eigenvectors of the quadratic form.^32,53–55^

To understand the sequence of pore filling during brine and surfactant injection quantitatively, an algorithm was employed to identify pore occupancy.^56–59^ This approach maps the pore space to a network of interconnected pores and throats, providing an enhanced representation of the pore structure. The phase occupying the center of each pore and throat was also identified at different stages of the displacement. Both curvature measurements and the computation of overall saturation were performed using Avizo 3D 2023.2., a commercial image analysis software.

## 3 Results and Discussion

In this section, we quantify pore-scale properties during tertiary surfactant flooding using high-resolution micro-CT images and compare the findings to previous work on secondary flooding.^31^ Section 3.1 visualizes and explores the oil displacement processes and proposes the oil recovery mechanism. Section 3.2 presents oil saturation profiles and recovery calculations. In Section 3.3, segmented images are analyzed to map fluid occupancy within the pore space. Wettability characterization follows in Section 3.4, where *in situ* contact angle measurements are examined alongside curvature analysis and capillary pressure calculations in Section 3.5. Finally, Section 3.6 investigates fluid connectivity.

### 3.1 Pore-Scale Displacement Processes and Proposed Mechanisms for Enhanced Oil Recovery

Figure 2 presents two-dimensional cross sections of the grayscale images, illustrating the sample at different stages of the experiment. These images were used to examine oil displacement within the pore network, focusing on a cross-sectional area of 1400 × 1400 μm^2^. Initially, when the sample was fully saturated with decane, prior to brine injection, the pores were predominantly filled with decane, with small brine droplets, as highlighted by the yellow circle in Figure 2A.

**Figure 2:**
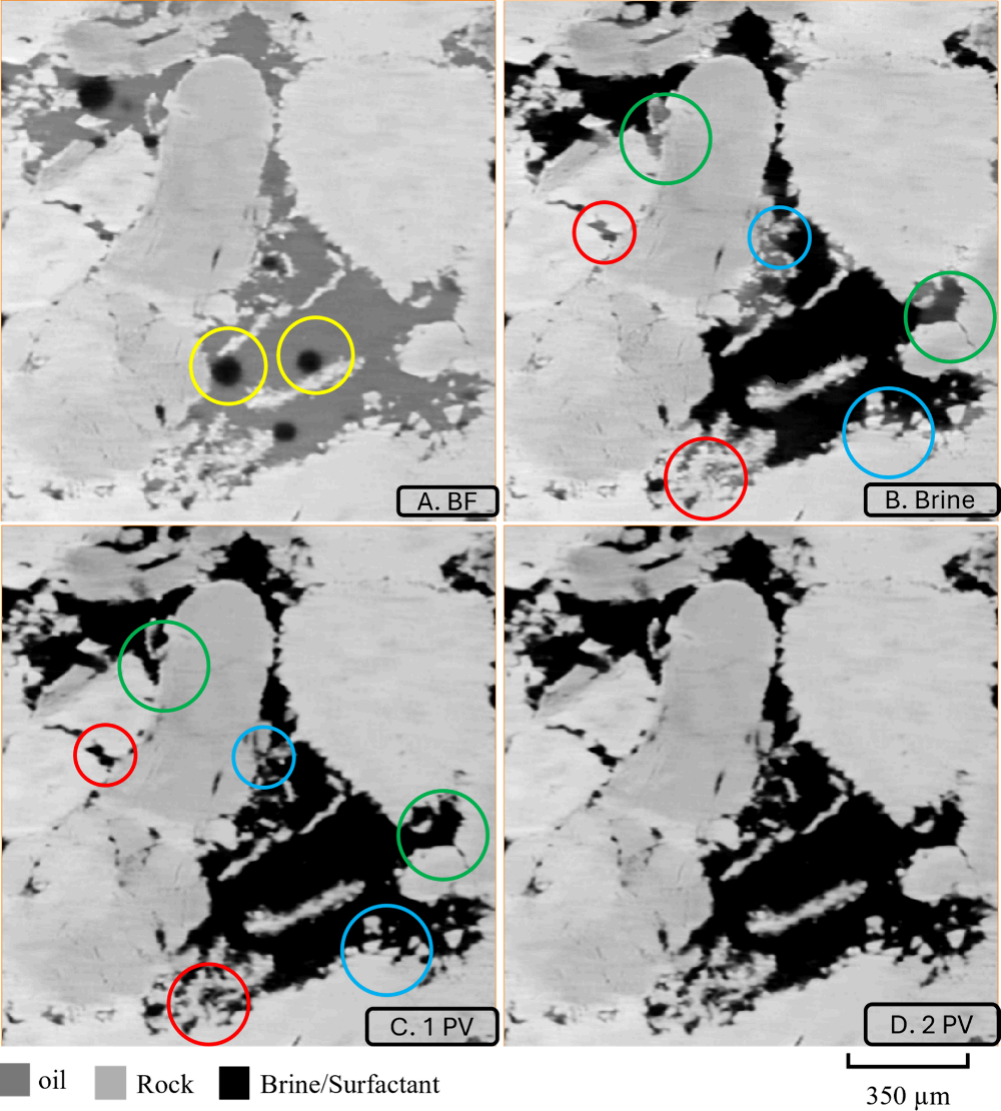
A cross-sectional view of the 2D grayscale images illustrating the distribution of oil and brine/surfactant within the pore space at different experimental stages. (A) (BF) Before flooding, oil occupied the majority of the pores, while brine was primarily confined to the central regions, often surrounded by oil. (B) (Brine) Following the injection of the first pore volume of brine, most oil in the pore centers was displaced, with residual oil remaining in the pore corners and smaller pores. (C) (1PV) After injecting the first pore volume of surfactant, a significant portion of the remaining oil was efficiently mobilized. (D) (2PV) No further significant oil displacement was observed after the injection of the second pore volume of surfactant, indicating that the maximum recovery had been achieved.

Following the injection of the first pore volume of brine, significant oil displacement occurred in the central regions of the pores. However, remaining oil remained in smaller pores (red circles), in dead-end pores (green circle), and at the pore corners (blue circle), as shown in Figure 2B. Subsequent injection of the first pore volume of surfactant led to further mobilization of oil from the smaller pores, as well as the oil in dead-end pores and pore corners, as shown in Figure 2C. No further oil displacement was observed after injecting the second pore volume of surfactant.

The enhanced oil recovery mechanism is attributed to surfactant-induced wettability alteration, accompanied by a moderate reduction in interfacial tension, as observed for secondary injection.^31^ This experiment involves a carbonate system primarily composed of calcite, brine enriched with the critical ions (Mg^2^^+^ and Ca^2^^+^), and oil containing both basic polar and significant acidic components. Therefore, we hypothesize that wettability alteration occurs in the following order. During brine saturation, divalent cations such as Mg^2^^+^ and Ca^2^^+^ preferentially adsorb onto the calcite surface, primarily via electrostatic attraction and surface complexation,^60^ while dissolution–precipitation processes may also contribute to ion equilibration. In the subsequent wettability alteration, the basic or acidic polar components in the oil are likely to adsorb onto the ion-equilibrated calcite surface through electrostatic interactions, hydrogen bonding, and van der Waals forces.^61,62^

Upon the injection of a surfactant, a typical cationic surfactant, wettability alteration is likely governed by two mechanisms. First, competitive adsorption between surfactant and basic polar oil components may displace the latter from the calcite surface.^15,63^ We consider that this mechanism has less impact on wettability alteration as the adsorption of surfactant to the rock surface is low as proved by multiple studies.^64–67^ Second, forming ion pairs between cationic surfactants and the negatively charged carboxylate ion that adsorbed onto calcite surfaces from crude oil^11,17,19,22^ and/or hydrophobic interactions between the nonpolar chains of oil molecules and surfactant aggregates promotes emulsification and reduces interfacial tension,^68^ thereby facilitating oil detachment and removal.

Importantly, these interpretations are consistent with surface complexation modeling, SCM, frameworks: Reviews highlight how SCMs capture calcite charging behavior,^69^ mechanistic models link competitive ion adsorption to wettability alteration in carbonates,^70^ and a recent study demonstrates that surfactant type strongly governs calcite/brine electrokinetics.^71^ Our results extend this framework by showing that cationic surfactants DTAB can also induce wettability alteration, most likely through adsorption of their positively charged headgroups to negatively charged calcite. We note that in our study, we did not directly measure surface charge or zeta potential; instead, the mechanism was inferred from pore-scale wettability observations, which are consistent with the electrokinetic interpretations from SCM studies.

### 3.2 Oil Saturation Profiles and Recovery

Using the segmented 3D images, Figure 3A shows the oil saturation distribution along the length of the sample. Initially, the oil is approximately uniformly distributed, with an original oil in place of approximately 87% and irreducible water saturation of around 13% in macro-pores, representing the baseline state of the sample, serving as a control for evaluating the effectiveness of subsequent injections. Brine injection results in a noticeable reduction in oil saturation, particularly near the inlet (*L* = 0 mm), demonstrating its effectiveness in mobilizing oil, as expected. Following this, surfactant flooding significantly enhances oil recovery by reducing interfacial tension and altering wettability, enabling better mobilization of residual oil, especially in areas less affected by brine. The surfactant’s impact is evident in the further reduction of oil saturation, overcoming brine injection limitations in mobilizing oil from smaller pores or regions of initially higher capillary pressure. Figure 3B presents the oil recovery achieved in the first half of the sample, measured from the inlet. This specific focus addresses the significant impact of the capillary end effect observed during the secondary flooding experiment.^31^ By analyzing only this section, we aim to ensure a fair comparison with previous work and effectively mitigate the influence of this phenomenon. The results show 15% incremental oil recovery after the first pore volume of surfactant injection and a minimal increase after the second pore volume. The experiment concludes with an ultimate recovery of 80%, highlighting the critical role of surfactants in maximizing the recovery. Comparing oil recovery between this experiment and secondary recovery^31^ shows that the first pore volume of surfactant injection in the secondary experiment resulted in 8% higher oil recovery than after brine injection alone in the tertiary experiment. Additionally, the ultimate recovery in the secondary experiment was 11% greater. The initial improvement in recovery is expected as surfactant flooding is more effective than water injection alone. That the ultimate recovery for secondary injection is higher implies that injecting in the tertiary mode is not optimal for local displacement efficiency: The injected surfactant tends to follow the flow paths followed by the initial water injection and does not contact as much of the remaining oil as when surfactant flooding is performed immediately.

**Figure 3:**
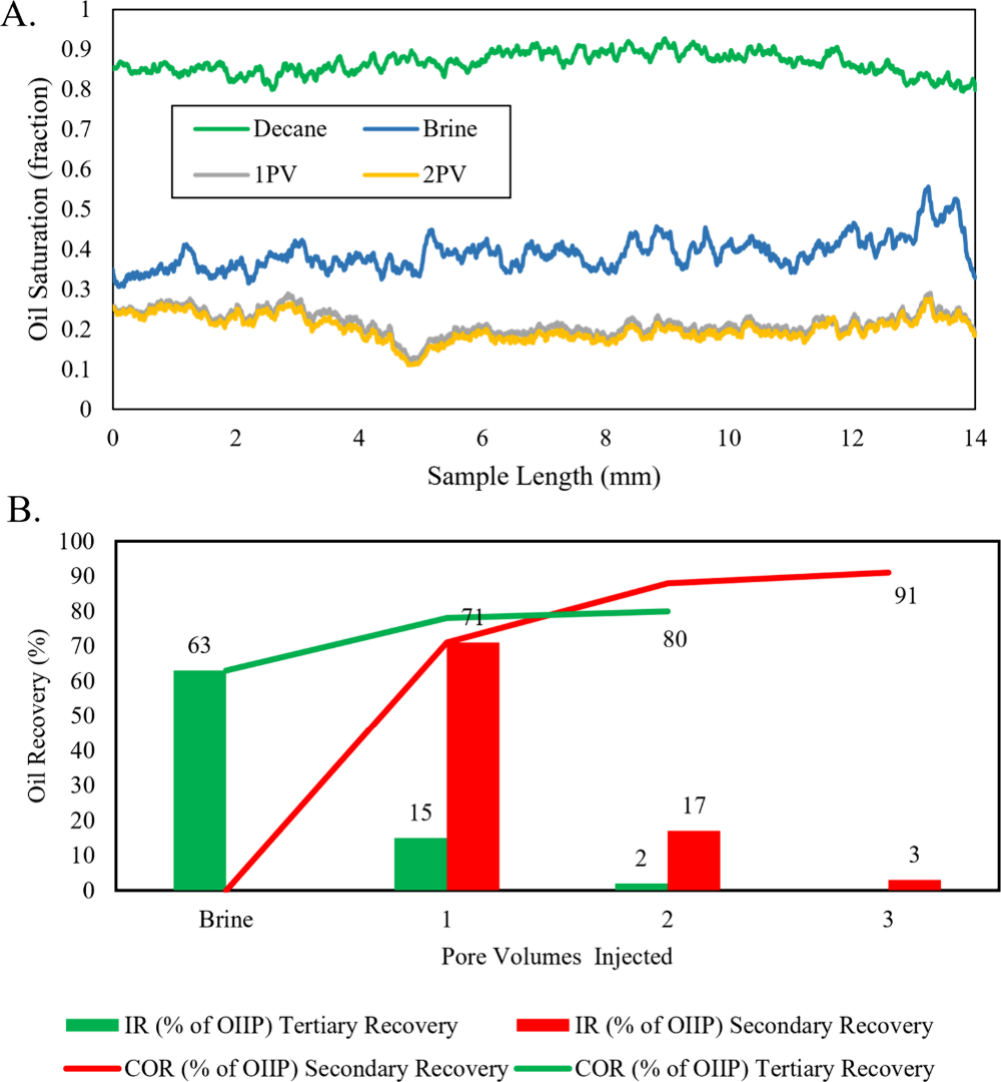
(A) Oil saturation along the sample length and after each pore volume injection. Initially, oil is uniformly distributed with an initial oil in place. OIIP, of 87%. (B) Comparison of cumulative oil recovery (COR) and incremental oil recovery (IR) after each injection stage as a percentage of the OIIP between the secondary [31] and tertiary experiments. To avoid capillary end effects, recoveries are shown for the first half of the sample. Brine injection improved oil recovery, while surfactant flooding further mobilized residual oil, achieving a 15% incremental recovery after the first pore volume and an ultimate recovery of 80% in the tertiary experiment. In the secondary experiment, surfactant recovered 71% of the oil after the first pore volume, with an additional 17% recovered after the second pore volume, resulting in an ultimate recovery 11% higher than that of the tertiary experiment.

### 3.3 Fluid Occupancy Mapping

Examining the fluid occupancy at the pore scale is vital for gaining insight into fluid distribution within the pore space and evaluating the impact of surfactant injection on the occupancy of pores and throats. A subvolume of 850 × 850 × 850 μm^3^ was extracted from the 3D segmented images of the rock sample’s center to investigate fluid distribution within pores and throats. Here, we illustrate the evolution of the fluid distribution at different flooding stages, highlighting changes in fluid occupancy and the efficiency of brine and surfactant injection. Figure 4A (top row) shows the probability density functions (PDFs) for pore and throat size distributions across all stages: before flooding (BF), after brine injection, and following the injection of one (1PV) and two pore volumes of surfactant (2PV). Initially, pore radii are distributed primarily in the ranges of 5–25 and 33–46 μm, while throat radii predominantly fall between 5 and 20 μm. Significant redistribution of fluids is observed following fluid injections, particularly after surfactant flooding.

**Figure 4:**
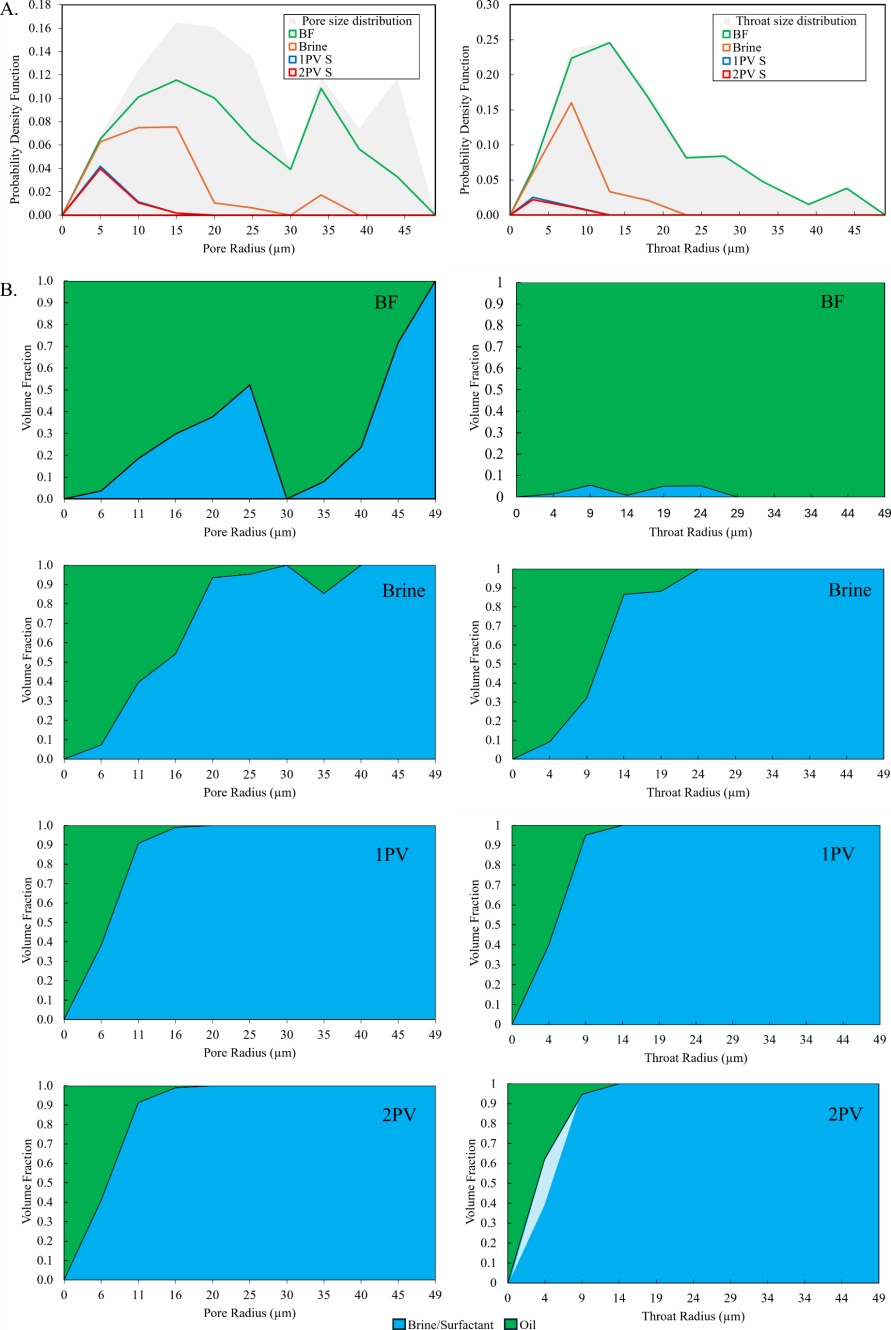
(A) Probability density functions of pore (left) and throat (right) size distributions before flooding (BF), after brine injection, and after one (1PV) and two pore volumes of surfactant injection (2PV). (B) Volume fractions of oil (green) and brine/surfactant (blue) in pores (left) and throats (right) for each stage, showing progressive oil displacement by brine and surfactant with residual oil confined to small pores and throats after 2PV. The pale-blue region in the probability density fraction for the throats after 2PV indicates the small amount of recovery due to surfactant injection beyond 1PV.

In Figure 4B, the first row (BF) represents the volume fractions of oil and brine in the pores and throats before flooding. Oil occupies most of small pores (radii less than 6 μm) and some medium size pores, while brine predominantly occupies the medium (radii between 10 and 30 μm) to large size pores (radii greater than 35 μm) and partially fills medium-sized throats (radii between 8 mm to 25 mm), reflecting an oil-wet system.^72^ After brine injection, shown in the second row, oil is displaced from medium and large pores (radii >10 μm) but remains largely in small pores and throats, indicating limited brine efficiency in altering the wettability of smaller pores and throats. The third row, corresponding to post injection of the first pore volume of surfactant (1PV), demonstrates the significant impact of surfactant flooding, as nearly all oil is displaced from medium and large pores, with a substantial reduction in oil content in small pores. This suggests effective mechanisms of interfacial tension reduction and wettability alteration by the surfactant. The final row, post injection of the second pore volume of surfactant (2PV), indicates that no significant further displacement of oil occurs, with minor oil displacement in throats as highlighted in light blue. We can see that residual oil confined mainly to small throats and some pores. This figure shows the progressive displacement of oil during brine and surfactant flooding and highlights the superior efficiency of surfactant in mobilizing oil compared to waterflooding alone.

### 3.4 Interfacial Area per Unit Volume

Fluid occupancy maps offer a simplified representation of fluid distribution, indicating the primary occupant of each pore. However, these maps do not account for fluids present in pore corners or in thin layers along pore walls. To address this limitation, the interfacial area per unit volume was calculated between solid and liquid (brine or surfactant solution, and oil) phases: oil–brine/surfactant (*A*_OB_), brine-rock (*A*_BR_) (defined before surfactant injection), surfactant-rock (*A*_SR_) (defined once surfactant has been injected), and oil-rock (*A*_OR_), as shown in Figure 5. Prior to surfactant flooding, the oil-rock interfacial area was large, indicating an oil-wet system where most surfaces were in contact with oil. Following the injection of the first pore volume of brine, the interfacial areas showed little change, indicating that there was no significant wettability alteration as expected: most of the oil remains near the walls of the rock sample and most of the recovered oil is from the middle of the pores.

**Figure 5:**
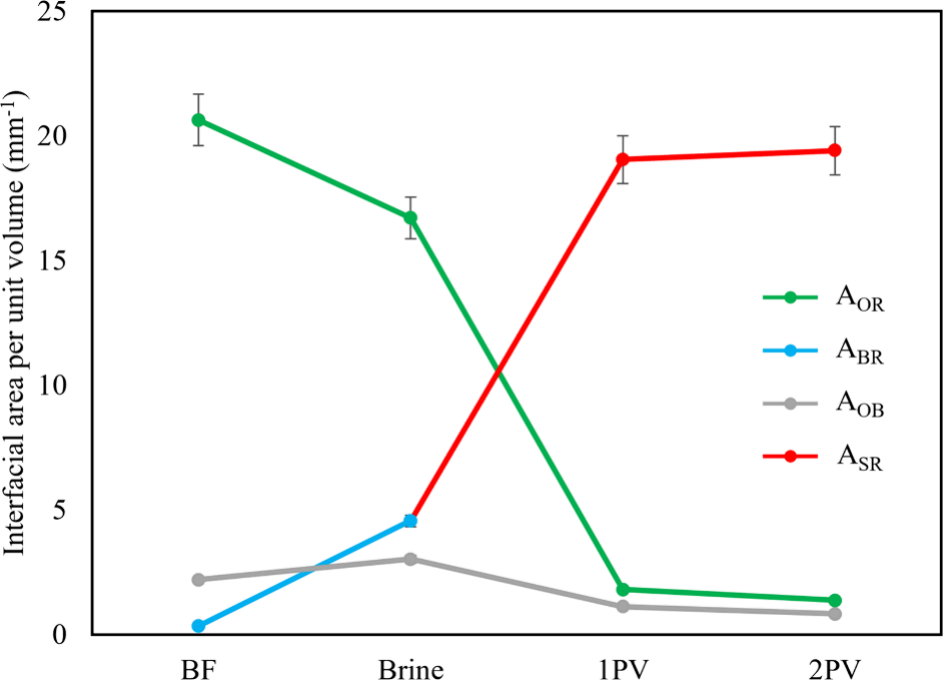
Interfacial area per unit volume, *A*, between rock R, oil O, and brine/surfactant B/S. The drop in *A*_OR_ after injecting the first pore volume of surfactant indicates a shift toward more water-wet conditions as now the aqueous phase preferentially contacts the solid.

Subsequent injection of the first pore volume of surfactant led to a substantial decrease in the oil-rock interfacial area and a big jump in surfactant-rock interfacial area indicating a wettability alteration which is responsible for 20% incremental oil recovery. After injecting the second pore volume of surfactant was injected, there was a decrease in both the oil-rock and oil-surfactant interfacial areas, reaching their respective minimum, while the surfactant-rock interfacial area increased to its maximum. This phenomenon can be attributed to the displacement of oil from smaller pores, allowing the surfactant to directly interact with the rock surface. Consequently, there is a shift in wettability, facilitating further mobilization of the oil. At the end of the displacement, since most of the oil is recovered, the oil-rock and oil-surfactant areas are small.

### 3.5 Contact Angle Distributions

Wettability alteration during waterflooding and surfactant flooding was evaluated by measuring *in situ* contact angles before and after each pore volume of brine and surfactant injection as illustrated in Figure 6A. The analysis focused on the spatial distribution of contact angles along the oil–water/surfactant-rock contact line within the same subvolume used for fluid occupancy mapping. Initially, before any flooding (BF), the sample exhibited a strongly oil-wet condition, with a mean contact angle of 124° and a standard distribution of 29°, indicating a wide range of local contact angle values. Figure 6B summarizes the statistics (the mean of all contact angles, the number of measured contact angles, and the standard deviation) for the contact angle calculations. Figure 6C,D shows a 2D cross section of the grayscale image and 3D visualization of a pore before any flooding and right after aging the sample where we can see the brine is bulging into oil, indicating an oil-wet system. After brine injection, the distribution of contact angle was similar with an average angle of 123° ± 25°, reflecting the continued oil-wet conditions of the sample. After injecting one pore volume of surfactant (1PV), a shift in wettability was observed, with the mean contact angle decreasing from 123 to 115° ± 22°. Although the change in the average contact angle appears modest there was a marked increase in local contact angles below 90°, indicating a shift toward more water-wet system. The second pore volume of surfactant (2PV) showed no additional change in the contact angle suggesting that the wettability alteration stabilizes after the first pore volume of surfactant injection.

**Figure 6:**
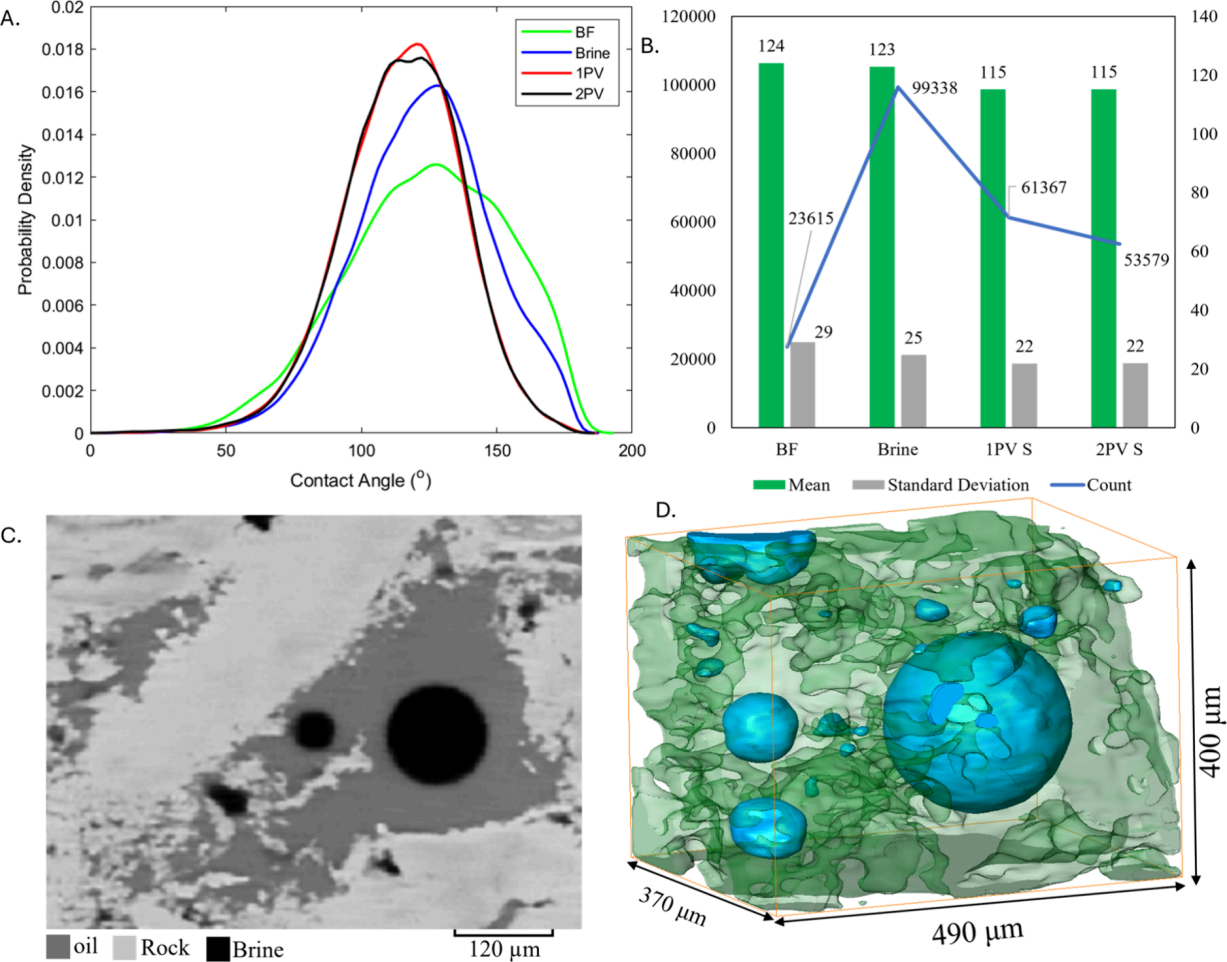
(A) Probability density functions of in situ contact angle measurements on the segmented 3D images at different flooding stages: before flooding (BF), after brine injection, and following one pore volume of surfactant (1PV) and two pore volumes of surfactant (2PV). The initial distribution (green line) shows a predominantly oil-wet system with contact angles above 90°. Following 1PV (red line), a significant shift toward mixed-wet conditions is observed. (B) Statistical summary of contact angle measurements. (C) Two-dimensional cross-sectional area of a grayscale image before flooding showing the brine droplet is surrounded by oil, an indication of initial oil-wet conditions. (D) Three-dimensional visualization of the same image.

### 3.6 Curvature and Capillary Pressure

The effect of waterflooding and surfactant flooding on rock wettability alteration, local capillary pressure, and fluid connectivity was assessed using curvature measurements at oil–brine/surfactant interfaces. The curvatures were estimated on the same subvolume of the high-resolution segmented images used in the analysis of fluid occupancy mapping. The interfaces between oil and brine/surfactant were smoothed, and two principal curvatures (κ_1_ and κ_2_) were extracted to calculate their mean, κ. The mean curvature was calculated using an interfacial area weighted average. Figure 7A is 3D visualization of the interfaces before and after surfactant flooding, showing noticeable changes indicating wettability alteration from an oil-wet system before surfactant injection to mixed wettability after surfactant injection. In addition, the distributions of mean curvature initially showed mostly negative mean curvature values, indicating oil-wet conditions with no major changes after injecting the brine, as shown in Figure 7B. However, once surfactant was introduced, the curvature distribution shifted, showing a broader mix of negative and positive values and moving the mean curvature toward the positive side, indicating a wettability change toward mixed-wet conditions.

**Figure 7:**
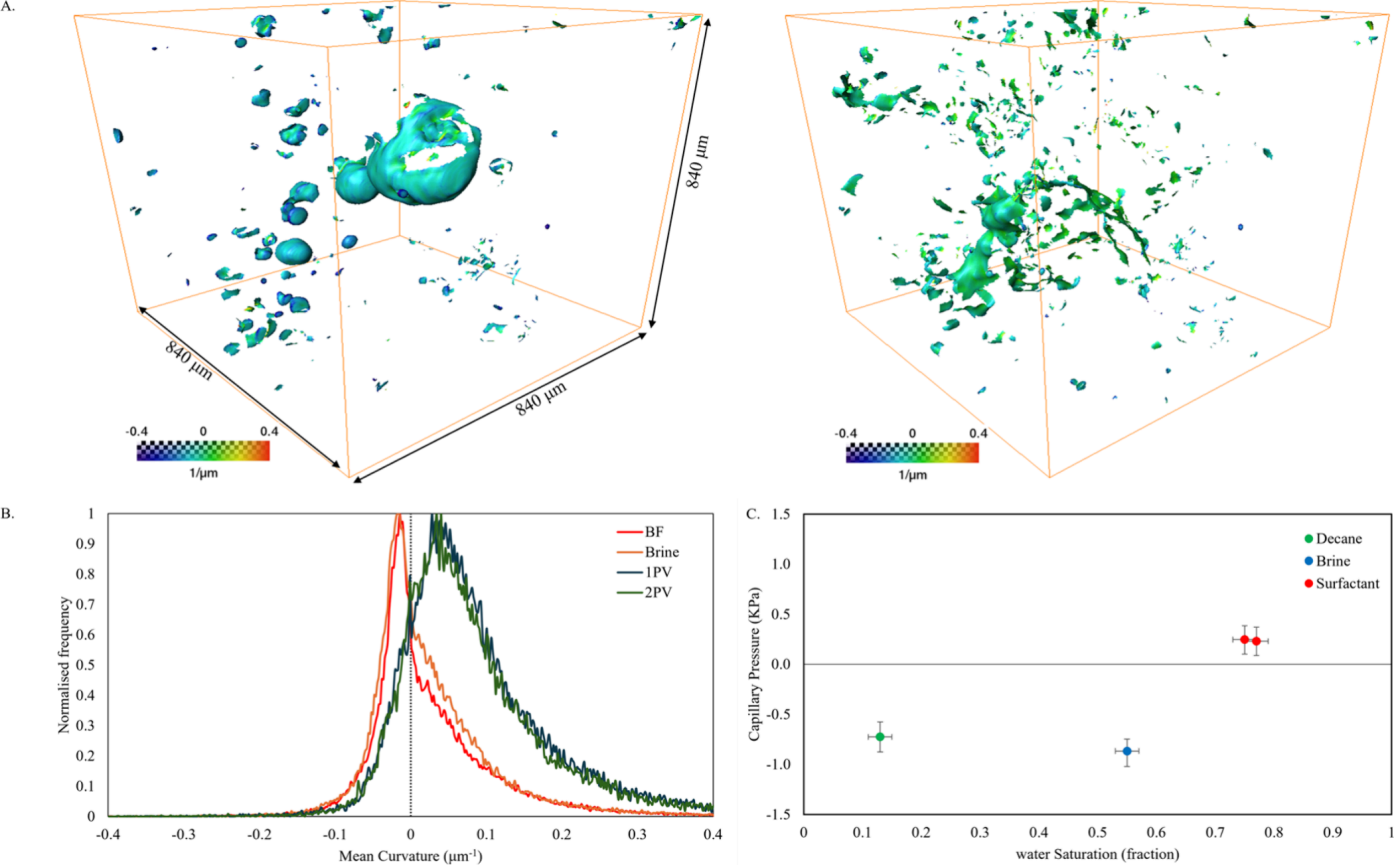
(A) 3D visualization of fluid–fluid interfaces indicating the mean curvature, with presurfactant flooding on the left and postsurfactant flooding on the right, color-coded by mean curvature values. (B) Histograms showing mean curvature variations before (BF) and after brine and surfactant flooding. (C) Capillary pressure estimates derived from mean curvature distributions (B) using eq 1, with uncertainties from image segmentation and the standard deviation of the distribution of curvature.

The local capillary pressure was determined by multiplying the average of the curvature distributions in Figure 7B and interfacial tension, using the Young–Laplace equation:^55,73,74^

$$P_{c}=2\sigma \kappa$$ 1

Here, σ denotes the interfacial tension between either oil–brine or oil–surfactant, while κ represents the average curvature from Figure 7B. Figure 7C shows the capillary pressure. Before surfactant injection, the capillary pressure was negative, confirming an oil-wet state that persisted through standard brine flooding. However, once a pore volume of surfactant entered the system, the capillary pressure shifted to positive values, illustrating a change in wettability and interfacial tension. Under normal circumstances, one would expect capillary pressure to drop as water saturation increases, but the opposite trend emerged once surfactant was introduced, which can only be explained by a wettability change. This trend aligns with previous findings in our work on secondary surfactant injection^31^ and those observed in low-salinity flooding.^75–77^ This outcome shows how surfactants can shift rock wettability and thereby enhance the displacement of oil.

### 3.7 Fluid Connectivity

Gaussian curvature (κ_1_κ_2_), derived from the product of the two principal curvatures (κ_1_ and κ_2_), can be used to assess fluid connectivity.^78,79^ Negative Gaussian curvature signifies well-connected phases, while positive values indicate poor connectivity or trapping of the nonwetting phase.^74,80^ For a more in-depth analysis of phase connectivity, the principal curvatures were classified into three distinct categories as shown in Figure 8, both positive (κ_1_ > 0, κ_2_ > 0) (shown in the blue histogram), both negative (κ_1_ < 0, κ_2_ < 0) (shown in the red histogram), and of opposite signs (κ_1_κ_2_ ≤ 0) (shown in the green histogram). The mean curvatures for the three groups were plotted against normalized frequency, as shown in Figure 8

**Figure 8:**
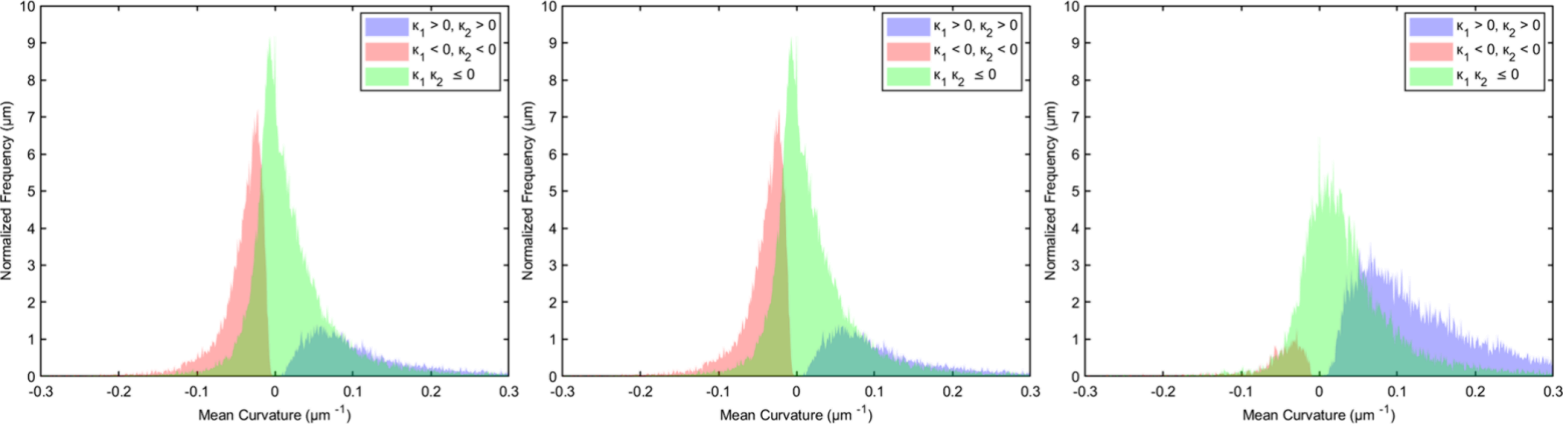
Mean curvature distributions of oil–brine/surfactant interfaces before flooding (left), after brine flooding (middle), and after surfactant flooding (right). Red, blue, and green areas indicate both curvatures negative (water bulging into oil), both curvatures positive (oil bulging into brine), and one positive and one negative curvature (saddle-shaped curvatures), respectively.

At first, the curvature distributions were mostly negative, suggesting oil-wet conditions and limited connectivity of the water phase within the pore space (left chart). The distributions of curvature were similar after brine injection. Following surfactant injection (right chart), there is a change with the rise in interfaces with one or two positive principal curvatures (shown in the blue and green histograms), suggesting a shift in wettability from oil-wet to more mixed-wet conditions. Fluid menisci with one positive and one negative curvature (green histogram) are more frequent than those with both positive menisci, the signature of a mixed-wet system and which, based on topological principles, leads to good connectivity of the oil and aqueous phases.^74,80,81,73^

## 4 Conclusions

This study provides a detailed pore-scale evaluation of tertiary surfactant flooding in a complex carbonate rock using high-resolution micro-CT imaging. Unlike our previous work on secondary surfactant flooding,^31^ which primarily focused on the early stages of surfactant injection, the current study explores the tertiary recovery stage, targeting oil that remains after secondary flooding has been completed. Our findings show that tertiary DTAB flooding can mobilize up to 77% of the initial oil through a combination of wettability alteration from an initial oil-wet state to a mixed-wet state associated with a moderate reduction in interfacial tension, demonstrating that this method can effectively recover oil from previously inaccessible pores and pore throats that were not mobilized by brine flooding alone.

The novel contributions of this study include the use of DTAB without cosolvents, offering a more economically viable alternative to surfactant systems that traditionally rely on ultralow interfacial tension for oil recovery. The study also provides insights into wettability alteration, interfacial tension reduction, and oil recovery mechanisms at the pore scale through advanced imaging techniques that have not been previously applied to tertiary surfactant flooding in carbonate reservoirs. These findings suggest that tertiary surfactant flooding is a promising EOR technique for mature reservoirs and offer a cost-effective solution for improving oil recovery efficiency in challenging geological formations.

Fluid occupancy and pore-scale displacement analysis using high-resolution images revealed a stepwise oil recovery process. Initially, surfactant displaced oil from medium and large pores (radii >15 μm), where brine flooding had already been partially effective. As injection progressed, additional oil was mobilized from smaller pores, but the smallest pores (radii <5 μm) remained largely oil-saturated, suggesting persistent capillary trapping even after surfactant flooding.

The analysis of contact angle and curvature measurements and interfacial area confirmed a shift in wettability from oil-wet to mixed-wet as also observed in secondary surfactant flooding.^31^ A transition in the capillary pressure was seen from negative to positive values that can only be explained by a change in wettability. Fluid connectivity analysis, based on Gaussian curvature measurements, confirmed that surfactant flooding significantly improved the connectivity of the phases, leading to a better displacement efficiency. Overall, however, the ultimate recovery from tertiary injection is 11% lower than a similar secondary experiment,^31^ indicating that beginning surfactant flooding early may have an advantage, allowing surfactant to contact more of the oil.

The carbonate rock surface was initially predominantly oil-wet due to the adsorption of polar components from crude oil onto the calcite surface.^61,62^ Upon DTAB injection, wettability alteration is driven by (i) competitive adsorption, where surfactant displaces basic polar oil components^15,63^ (although this is considered less important due to low surfactant adsorption^64–67^ ), and (ii) ion pair formation between DTAB and negatively charged carboxylates adsorbed from crude oil,^11,17,19,22^ and/or hydrophobic interactions between oil and surfactant aggregates,^50^ which promote emulsification, reduce interfacial tension, and facilitate oil detachment.

These results demonstrate that DTAB can enhance oil displacement in carbonate formations without requiring ultralow interfacial tension, making it a cost-effective alternative for improving the recovery efficiency in complex carbonate reservoirs. The findings highlight the importance of wettability alteration and improved fluid connectivity in maximizing oil mobilization.

Future work will focus on conducting additional tertiary surfactant flooding experiments at surfactant concentrations close to the critical micelle concentration (CMC). Comparing the performance of DTAB injection above and near the CMC will help clarify the role of concentration in the wettability alteration and recovery efficiency. Furthermore, the measurement of relative permeabilities (*k*_rw_/*k*_ro_ vs *S*_w_) for both brine and surfactant flooding will provide valuable quantitative insight into how surfactant alters flow functions compared to conventional waterflooding. Such studies will allow a more comprehensive evaluation of the efficiency of surfactant flooding in carbonate reservoirs
